# Validity and reliability of the Turkish version of the peace, equanimity, and acceptance in the cancer experience (PEACE) scale

**DOI:** 10.1017/S1478951525100588

**Published:** 2025-08-05

**Authors:** Ömer Ödek, Mumin Savaş, Hakan Çelik, Gönül Taşcı, Ender Doğan, Handan Zincir

**Affiliations:** 1 Ministry of Health, Kayseri City Training and Research Hospital, Kayseri, Turkey; 2 Department of Public Health Nursing, Faculty of Health Sciences, Adıyaman University, Adıyaman, Türkiye; 3 Department of Public Health Nursing, Faculty of Health Sciences, Çukurova University, Adana, Turkey; 4 Erciyes University Health Sciences Institute, Kayseri, Turkey; 5 Department of Public Health Nursing, Faculty of Health Sciences, Erciyes University, Kayseri, Turkey

**Keywords:** Cancer, acceptance, PEACE Scale, reliability, validity

## Abstract

**Objectives:**

This study sought to examine the validity and reliability of the Turkish adaptation of the Peace, Equanimity, and Acceptance in the Cancer Experience (PEACE) scale. The primary objective was to evaluate the scale’s psychometric properties in measuring acceptance and coping among cancer patients.

**Methods:**

The study included 90 cancer patients who completed the 12-item PEACE scale. The scale consists of two distinct subscales: the 5-item Peaceful Acceptance subscale and the 7-item Struggle With Illness subscale. Reliability was examined using Cronbach’s alpha and test-retest reliability (*r* = 0.916). Content validity was assessed using the content validity index (CVI = 0.84). Both exploratory factor analysis (EFA) and confirmatory factor analysis (CFA) were employed to examine the underlying factor structure and evaluate model fit indices.

**Results:**

The internal consistency for both subscales was satisfactory (Cronbach’s α = .78 for both). EFA indicated that the two subscales explained 53.169% of the total variance. CFA substantiated the two-factor model, demonstrating adequate model fit indices (χ^2^/df = 1.689,Root Mean Square Error of Approximation = 0.088). These findings collectively establish the Turkish version of the PEACE scale as a psychometrically sound tool.

**Significance of Results:**

The PEACE scale is a valid and reliable instrument for assessing levels of acceptance and coping in cancer patients. Its use can help healthcare professionals better understand patients’ emotional states and guide interventions aimed at improving their quality of life.

## Introduction

Cancer is a life-threatening disease, and many distressing situations arise in patients following its diagnosis. Many cancer patients undergo intensive treatment with significant symptom burden, functional limitations, and financial difficulties (Cleeland et al., 2016; Mosher et al., 2021). In advanced stages of cancer, patients often face limited life expectancy, complex medical decision-making, and end-of-life planning situations. Approximately one-third of cancer patients suffer from mood disorders or clinically high levels of distress, including increased anxiety and depressive symptoms (Hassan et al., 2015; Unseld et al., 2019). Eighty percent of patients experience cancer-related post-traumatic stress, decreased quality of life, and poor compliance with treatment (Unseld et al., 2019).

Acceptance in the context of cancer is an emotion-focused coping mechanism that involves accepting the reality of the disease, learning to live with it, and making attempts to address it (Pinquart et al., 2006). Cancer patients must accept the disease and live at peace with it in order to cope with the associated challenges (Quinto et al., 2022). It is believed that an in-depth measurement of peace with cancer in patients and increasing levels of peace among patients with low acceptance will contribute positively to their fight against the disease (Chen et al., 2019; Mack et al., 2008). Czerw et al. concluded that individuals who highly accepted their cancer diagnosis experienced lower levels of anxiety and hopelessness (Czerw et al., 2017).

The significance of acceptance for cancer patients can be further elucidated through studies examining the impact of supportive interventions that incorporateacceptance-based approaches, such as Acceptance and Commitment Therapy (ACT), mindfulness, and meaning-centered interventions. Research has consistently shown that acceptance contributes to lower psychological distress, better emotional regulation, and improved quality of life among cancer patients (Feros et al., 2013; Rost et al., 2012). For instance, ACT-based interventions have been associated with reduced anxiety, depression, and increased psychological flexibility in individuals coping with advanced-stage illness (Hayes et al., 2006). These findings underscore the importance of assessing and promoting acceptance as a therapeutic target in psycho-oncology. In the context of Turkish society, acceptance is a multifaceted concept often shaped by religious beliefs, collectivist family values, and societal norms surrounding illness and suffering. In many cases, spiritual surrender, belief in destiny, and reliance on familial care systems can facilitate acceptance and peaceful coexistence with illness (Akyüz and Erdem, 2013; Karahan et al., 2018). However, these cultural values may also create inner conflicts, especially when individuals feel they must remain “strong” for their families or when emotional expression is socially restrained. Therefore, understanding what acceptance means within Turkish cultural and spiritual frameworks is crucial. Factors such as religious coping, perceived social support, fatalistic beliefs, and prior exposure to illness within the family may all influence an individual’s capacity to accept their diagnosis (Koç and Sağkal, 2020).

Clarifying the meaning and determinants of acceptance in this sociocultural context can help tailor psychosocial interventions more effectively. It also strengthens the rationale for validating tools like the PEACE scale, ensuring that they capture culturally relevant expressions of acceptance and struggle. Ultimately, this approach aligns with culturally sensitive care models, aiming to provide more meaningful support to patients facing life-threatening conditions.

In 1988, Merle Mishel developed the “Uncertainty Theory in Diseases” to define and explain the uncertainty that patients often experience, making it applicable to nursing practice and research (Mishel 1990). The theory explains how individuals find meaning in illnesses. It aims to provide a realistic understanding of how cancer and chronic diseases create imbalance and how individuals adapt to uncertainty to find new meaning in the disease. According to the model, reducing uncertainty, especially in cancer patients, is considered one of the most critical factors in battling the disease. Individuals experiencing uncertainty during their illness are at high risk for difficulties. Acceptance of the disease and coping abilities are among the most significant uncertainties in cancer patients (Ahadzadeh and Sharif, 2018; Sharif, 2017).

The Peace, Equanimity, and Acceptance in Cancer Experience (PEACE) scale was developed to assess peaceful acceptance in the fight against cancer, a terminal illness (Mack et al., 2008). The scale comprises two subdimensions: peaceful acceptance of the disease and struggle with the disease. It measures the extent to which patients accept their cancer diagnosis and struggle with the disease (Mack et al., 2008). Cultural differences may influence how cancer is accepted and how patients struggle with the disease. While some cultures emphasize religious coping, prayer, and reconciliation with God, others emphasize the perception of fighting cancer (Tang et al., 2019). A review of the literature indicates that, although there is no psychometrically validated scale specifically developed to assess cancer-specific acceptance levels among Turkish patients, several studies have examined illness acceptance using general illness acceptance scales. For instance, Çevik et al. (2022) investigated illness acceptance and hope levels in cancer patients by employing a generic illness acceptance scale, rather than one specifically designed to capture the unique psychological and existential aspects of the cancer experience. This reveals a significant gap in the availability of culturally adapted, diagnosis-specific instruments for assessing acceptance and coping among cancer patients in Turkey. Therefore, this study aims to validate and assess the reliability of the Turkish version of the Peace, Equanimity, and Acceptance in Cancer Experience (PEACE) scale, aiming to offer a culturally relevant tool that accurately measures cancer-specific acceptance and struggle in the Turkish population.

### Research questions

1. Is the Peace, Equanimity, and Acceptance in the Cancer Experience (PEACE) Scale a reliable and valid measurement tool in Turkish?

2. Are the dimensions of the Peace, Equanimity, and Acceptance in the Cancer Experience (PEACE) Scale associated with sociodemographic variables?

## Methods

### Design and sample

This methodological study was conducted to examine the psychometric properties of the PEACE scale. The study population consisted of cancer patients receiving chemotherapy treatment and being followed at the oncology outpatient clinic of Kayseri City Training and Research Hospital in Kayseri, Turkey. The sample comprised 90 cancer patients who were randomly selected from the study population and agreed to participate. The inclusion criteria were as follows: being 18 years or older, having no loss of consciousness or mental disability, and being able to read and write in Turkish. The sample size in this study was determined based on methodological recommendations for scale adaptation and psychometric evaluation, specifically adhering to the widely accepted rule of thumb recommending a minimum of 5 to 10 participants per item on the scale (Osborne and Costello, 2004). Since the PEACE scale consists of 12 items, a minimum sample size of 60 to 120 participants was required. In our study, a total of 90 participants were recruited, which satisfies these methodological criteria and ensures adequate statistical power for factor analysis and reliability testing. Although the original validation study of the PEACE scale included 160 participants, sample size requirements can vary depending on the context of the study, and the current sample size is sufficient according to the accepted standards in the literature. Considering these values, it can be said that a sample size of 100 individuals is sufficient for the 20-question test used in this study. In addition, the KMO value of the test was 0.647, and Bartlett’s test was found to be significant (*p* < .05). Based on the results of the KMO and Bartlett’s tests, it was determined that the sample size and the normality of the data distribution were adequate to proceed with with factor analysis.

### Procedure

Linguistic Validity: In this study, the form developed by the World Health Organization was translated and adapted into Turkish. The translation of the PEACE scale from English to Turkish was independently conducted by a linguist fluent in both languages. To detect potential inaccuracies and inconsistencies in the translation process, the scale was back-translated from Turkish to English by 10 experts who held at least a doctoral degree and had advanced proficiency in both Turkish and English. Item-by-item comparisons were made to ensure conceptual and linguistic appropriateness, and the accuracy of the translation was evaluated by an expert (the third author) using the back-translation method to verify its consistency with the original text.

In addition, the Davis technique was employed to assess the content validity of the Turkish version. Experts were asked to evaluate each item in terms of relevance, clarity, and cultural appropriateness using a four-point scale. The content validity index (CVI) was then calculated based on these ratings. Items with low scores were revised or removed according to expert feedback.

Experts examined the content validity of the PEACE scale, and necessary modifications were made to the Turkish version. Each modification was carried out in line with the evaluations of the experts. To test the validity of the data collection tools, a pilot study was conducted with 10 participants who were not included in the research sample. Based on the suggestions from the pilot study, necessary corrections were made, and the final version of the data collection tool was applied to the sample group. The process of cultural adaptation of the scale was not limited to linguistic translation alone; it also took into account the perception of illness, death, and salvation within Turkish society. During the translation-back-translation process, it was examined whether the scale maintained its semantic integrity, and no modifications were deemed necessary due to culturally specific illnesses. In particular, while the term “acceptance” could carry different connotations in Turkish, additional evaluations were conducted to assess how the Turkish population might interpret it.

### Data collection tool

In this study, the “Demographic Characteristics of the Participants Form,” along with the Peace, Equanimity, and Acceptance in Cancer Experience (PEACE) Scale and the Cancer-Related Negative Social Expectations Scale (CRNSES) developed by Mack et al. (2008), were used as data collection tools. The Descriptive Information Form comprises nine questions created by the researcher based on literature knowledge to reveal the demographic characteristics, occupational details, and thoughts about death among the study participants (Okamura et al., 2022). The PEACE Scale, developed by Mack et al. (2008), consists of 12 items and employs a Likert scale. The lowest possible score on the scale is 12, while the highest is 48. An increase in the score indicates a higher level of cancer acceptance among patients, whereas a decrease in the score signifies a lower level of acceptance. The Cronbach’s alpha coefficient for the PEACE Scale is 0.85 (Mack et al., 2008). The Cancer-Related Negative Social Expectations Scale (CRNSES) was developed as a 5-item scale based on loneliness theory and previous studies. It was validated for Turkish reliability by Kara and İ. (2020). The scale assesses patients’ negative social cognitions about their cancer experiences, utilizing a 6-point Likert-type rating scale. The Cronbach’s alpha value for the scale is 0.90 (Kara and İ., 2020).

### Data collection and ethical issues

Data were collected from cancer patients who voluntarily participated in the study through face-to-face interviews. Completing the data collection instrument took approximately 20–25 minutes per participant. Following the initial data collection, the instrument was re-administered to 30 participants from the same sample group who consented to participate in the test-retest phase. The study employed a descriptive and methodological research design. Ethical approval was granted by the Erciyes University Social and Humanities Ethics Committee (31 January 2023, Approval No. 05).

### Data analysis

SPSS 22.0 and LISREL 8.7 programs were used for the validity and reliability analyses required for the development of the scale. In order to evaluate the construct validity of the scale, KMO and Bartlett’s test analyses were conducted to determine the appropriateness of the data for factor analysis procedures. Exploratory factor analysis was performed based on the data obtained. After the exploratory factor analysis, confirmatory factor analysis was performed. Internal consistency coefficients were analyzed to determine the reliability of the scale. Cronbach’s Alpha reliability coefficient was calculated to determine the internal consistency level. Statistical analyses were conducted to examine the associations between patient characteristics and the PEACE subscales (Peaceful Acceptance and Struggle with Illness). Independent samples t-tests were used to compare two-group variables (e.g., gender, marital status, living alone, chemotherapy status, and recurrent cancer/metastasis). One-way analysis of variance (ANOVA) was performed for variables with three or more categories (e.g., age groups and education level). Post-hoc tests (e.g., Tukey HSD or Bonferroni) were applied when ANOVA results indicated significant differences. A significance level of *p* < 0.05 was considered.

## Results

### Participants’ characteristics

The study sample consisted of 90 participants, comprising 49 (54.4%) women and 41 (45.6%) men. Examination of the age distribution revealed that 32.2% (n = 29) of participants were under 39 years of age, 45.6% (n = 41) were between 40 and 64 years of age, and 22.2% (n = 20) were 65 years and older. Demographic data are given in Table 1.

**Table 1.** Demographic characteristics of the participants (n = 90)

Variable	Categories	Number of People (n)	Percentage (%)
Age	Under 39	29	32.2
	40 − 64	41	45.6
	65 and above	20	22.2
Gender	Female	49	54.4
	Male	41	45.6
Marital Status	Married	69	76.7
	Single	21	23.3
Education	Primary-Secondary School	52	57.8
	High School	17	18.9
	Undergraduate	16	17.8
	Postgraduate	5	5.6
Living Alone	Yes	11	12.2
	No	79	87.8
Type of Cancer	Lung	23	25.6
	Prostate	5	5.6
	Breast	13	14.4
	Colon	11	12.2
	Uterus	3	3.3
	Thyroid	4	4.4
	Stomach	4	4.4
	Liver	8	8.9
	Leukemia	3	3.3
	Kidney	2	2.2
	Brain	8	8.9
	Pancreas	1	1.1
	Other	5	5.6
Currently Receiving Chemotherapy	Yes	45	50.0
	No	45	50.0
Recurrent Cancer or Metastasis	Yes	42	46.7
	No	48	53.3

## Validity testing

### Content validity

The final version of PEACE was submitted to five subject matter experts, who were briefed on the instrument’s purpose and underlying theoretical constructs. The experts were asked to rate the necessity of each PEACE item on a five-point Likert scale. The content validity index (CVI) of the scale was calculated as 0.84. Given that this value exceeded the acceptable threshold of 0.70 for item-level content validity, the PEACE scale demonstrated satisfactory content validity in this investigation.

### Construct validity

In the factor analysis, the principal component extraction method was applied using varimax rotation. Bartlett’s test of sphericity yielded a value of 421.657 (*p* < 0.001), indicating that the correlations among items were sufficient for factor analysis. The Kaiser-Meyer-Olkin (KMO) measure of sampling adequacy was 0.760, which falls within the acceptable range (greater than 0.60). Following these preliminary checks, exploratory factor analysis (EFA) was conducted. According to the scree plot, two factors with eigenvalues greater than 1.0 were identified, aligning with Kaiser’s criterion for factor retention. Factor 1, called the “Peaceful Acceptance subscale,” consists of 5 items (items 1–5) and explains 31.944% of the variance with an eigenvalue of 3.833. Factor 2, called the “Struggle With Illness subscale,” consists of 7 items (items 6–12) and explains 21.225% of the variance with an eigenvalue of 2.547. The factor loadings of all items were higher than 0.30, which meets the commonly accepted threshold for meaningful factor loadings Table 2.

**Table 2.** Construct validity of the PEACE: individual item loadings from the exploratory factor analysis (n = 90)

Items	Factor 1	Factor 2	Total item correlation	Mean	Standard Deviation (SD)	Corrected Item-Total Correlation	Cronbach’s Alpha if Item Deleted
1. To what extent are you able to accept your diagnosis of cancer?	0.173	**0.703**	0.497	2.96	0.82	0.315	0.567
2. To what extent would you say you have a sense of inner peace and harmony?	−0.207	**0.838**	0.380	2.63	0.76	0.105	0.610
3. To what extent do you feel that you have made peace with your illness?	−0.178	**0.771**	0.347	2.69	0.80	0.150	0.602
4. Do you feel well loved now?	0.116	**0.575**	0.368	3.39	0.63	0.217	0.587
5. To what extent do you feel a sense of inner calm and tranquility?	−0.263	**0.625**	0.340	2.68	0.63	−0.006	0.623
6. To what extent do changes in your physical appearance upset you?	**0.760**	0.344	0.719	3.33	0.83	0.588	0.499
7. To what extent does worrying about your illness make it difficult for you to live from day to day?	**0.785**	0.082	0.612	3.34	0.80	0.460	0.534
8. To what extent do you feel that it is unfair for you to have cancer now?	**0.592**	−0.452	0.353	2.67	0.79	0.159	0.600
9. To what extent do you feel that your life, as you know it, is now over?	**0.632**	−0.428	0.419	2.62	0.89	0.206	0.593
10. To what extent do you feel angry because of your illness?	**0.482**	−0.310	0.359	1.97	0.66	0.200	0.590
11. To what extent do you think your illness has beaten you down?	**0.833**	0.086	0.672	3.49	0.66	0.563	0.523
12. To what extent do you feel ashamed of or embarrassed by your current condition?	**0.415**	−0.155	0.341	2.52	0.77	0.153	0.601
Eigenvalue	3.833	2.547					
Percent total variance	31.944	21.225					

**Note**: ExtractionMethod: Principal Component Analysis. Rotation Method: Varimax with Kaiser Normalization. Rotation converged in 5 iterations.

**Figure 1.**
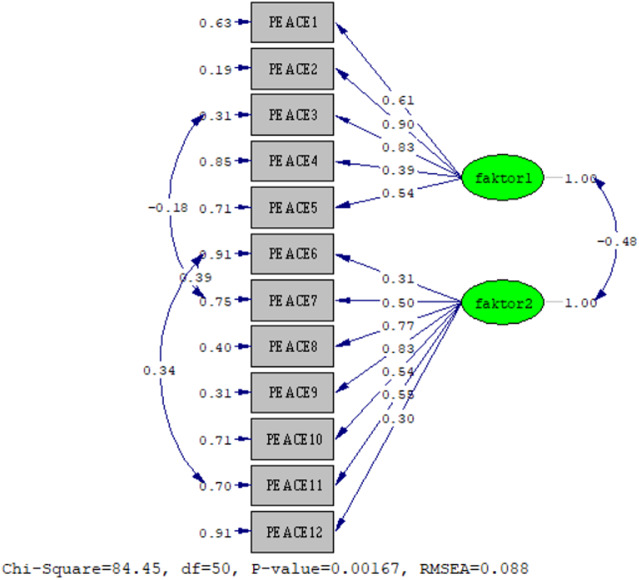
Path diagram.

The factor structure obtained by EFA was analyzed using confirmatory factor analysis (CFA) with LISREL 8.7. Figure 1 shows the final model obtained after examining the modification indices related to model incompatibility. PEACE demonstrated acceptable fit indices in the CFA with 12 items and two factors (χ² (N = 90) = 84.45, *p* < 0.001; χ²/df = 1.689), where values of χ²/df below 3.0 indicate good model fit. The fit indices (CFI = 0.93, NFI = 0.93, NNFI = 0.91, IFI = 0.93, RMSEA = 0.088) were within acceptable ranges, with CFI, NFI, NNFI, and IFI all above 0.90, and RMSEA indicating a mediocre fit (0.08–0.10) Table 3.

**Table 3.** Fit indices of the five-component structure model of the PEACE

Fit Index	Value	Normal (Acceptable) Range
Comparative Fit Index (CFI)	0.93	≥ 0.90 (good fit), ≥ 0.95 (excellent fit)
Normed Fit Index (NFI)	0.93	≥ 0.90 (acceptable)
Non-Normed Fit Index (NNFI)	0.91	≥ 0.90 (acceptable), ≥ 0.95 (good)
Incremental Fit Index (IFI)	0.93	≥ 0.90 (acceptable)
Root Mean Square Error of Approximation (RMSEA)	0.088	≤ 0.05 (good fit), 0.05 − 0.08 (acceptable), 0.08 − 0.10 (mediocre), > 0.10 (poor fit).

As presented in Table 4, a weak negative correlation was observed between Factor 2 and Factor 1, while Factor 2 exhibited strong positive correlations with both CRNSES and PEACE total scores (*r* = − 0.243, *r* = 0.648, and *r* = 0.748, respectively; *p* < 0.05). Factor 1 demonstrated a weak negative correlation with CRNSES and a moderate positive correlation with the PEACE total score (*r* = − 0.227 and *r* = 0.462, respectively; *p* < 0.01). Additionally, a moderate positive correlation was observed between CRNSES and PEACE scores (*r* = 0.438, *p* < 0.01). This moderate correlation suggests that individuals who report higher levels of acceptance and inner peace in the face of cancer may also perceive greater negative social expectations or isolation. One possible interpretation is that, in the context of Turkish culture, where family and societal expectations are central, patients may develop internal peace as a compensatory coping mechanism when social support is lacking. Alternatively, those with higher inner awareness may also be more perceptive of societal stigma or negative expectations. These findings support the concurrent validity of the Turkish PEACE scale and emphasize the complex interplay between individual and social dimensions of coping in cancer patients.

**Table 4.** Correlation matrix and significance levels between PEACE and CRNSES total scores (<0.001)

Scale	PEACE Factor 2	PEACE Factor 1	CRNSES Total Score	PEACE Total Score
PEACE Factor 2	–	−0.243*	0.648**	0.748**
*p*		0.021	**0.001**	**0.001**
PEACE Factor 1		–	−0.227**	0.462**
*p*			**0.001**	**0.001**
CRNSES Total Score			–	0.438**
*p*				**0.001**
PEACE Total Score				–

**Note.** *PEACE* = Peace, Equanimity, and Acceptance in the Cancer Experience Scale; *CRNSES* = Cancer-Related Negative Social Expectations Scale. Correlation coefficients (*r*) are shown above the diagonal. *p < 0.05; **p < 0.01; *p*-values are two-tailed.

### Reliability testing

Cronbach’s coefficient was calculated to determine the internal consistency of PEACE, and the Cronbach's value of the scale fell within the acceptable value range. The Cronbach's coefficient for the whole scale was 0.601, and the sub-dimension Cronbach's values were calculated as 0.784 and 0.786 for the sub-dimensions, respectively (Table 5). The test-retest reliability of the scale was analyzed two weeks after the first application, and the scale was reapplied to 30 cancer patients. The test-retest value of the scale was calculated as 0.916, which shows that the scale is reliable.

**Table 5.** Internal consistency of the PEACE (Cronbach’s α Coefficient, N = 90)

Subscales (no of items)	Composite scores (Mean ± SD)	Cronbach’s α coefficient
Factor 1 (5 items) – Peaceful Acceptance	14.34 ± 2.68	0.783
Factor 2 (7 items) – Struggle with Illness	19.94 ± 3.58	0.784

**Note**: Cronbach’s alpha values above 0.70 indicate acceptable internal consistency, and values above 0.80 indicate good internal consistency. Therefore, both subscales demonstrated satisfactory internal consistency.

### Associations between patient characteristics and the PEACE subscales

According to Table 6, several sociodemographic and clinical characteristics were significantly associated with the PEACE Scale subdimensions and total score.

**Table 6.** Associations between patient characteristics and the PEACE subscales (n = 90)

Demographic characteristics		N (%)	Factor 1: Peaceful Acceptance (X ± SD)	*p*	Factor 2: Struggle with Illness X ± SD	*p*	PEACE total score X ± SD	*p*
Age	Under 39	29(%32.2)	15.03 *±* 2.27	0.165	20.68 *±* 2.97	0.364	35.72 *±* 3.42	0.054
	40 − 64	41(%45.6)	13.80 *±* 2.82		19.73 *±* 4.20		33.53 *±* 3.88	
	65 and above	20(%22.2)	14.45 *±* 2.81		19.30 *±* 2.93		33.75 *±* 4.27	
Gender	Female	49(%54.4)	14.73 *±* 2.88	0.118	19.40 ± 3.14	0.189	34.14 ± 3.94	0.702
	Male	41(%45.6)	13.87 ± 2.36		20.58 ± 3.99		34.46 ± 3.93	
Marital Status	Married	69(%76.7)	14.18 ± 2.86	**0.045**	19.79 ± 3.70	0.930	33.98 ± 3.91	0.185
	Single	21(%23.3)	14.85 ± 1.95		20.42 ± 3.18		35.28 ± 3.86	
Education	Primary-Secondary School	52(%57.8)	14.03 ± 2.49	**0.010**	19.84 ± 3.31	0.104	33.88 ± 3.52	**0.001**
	High School	17(%18.9)	13.29 ± 3.15		19.47 ± 4.59		32.76 ± 4.53	
	Undergraduate	16(%17.8)	16.00 ± 2.06		21.56 ± 2.50		37.56 ± 2.75	
	Postgraduate	5(%5.6)	15.80 ± 2.28		17.40 ± 4.33		33.20 ± 4.08	
Living Alone	Yes	11(%12.2)	13.45 ± 2.73	0.242	21.00 ± 2.68	0.300	34.45 ± 2.46	0.882
	No	79(%87.8)	14.46 ± 2.66		19.79 ± 3.68		34.26 ± 4.09	
Currently Receiving Chemotherapy	Yes	45(%50.0)	14.26 ± 2.65	0.785	20.80 ± 3.32	**0.023**	35.06 ± 4.11	0.059
	No	45(%50.0)	14.42 ± 2.73		19.08 ± 3.67		33.51 ± 3.59	
Recurrent Cancer or Metastasis	Yes	42(%46.7)	13.85 ± 2.72	0.107	21.38 ± 2.74	**0.001**	35.23 ± 3.68	**0.031**
	No	48(%53.3)	14.77 ± 2.59		18.68 ± 3.78		33.65 ± 3.96	

Marital status and education level showed statistically significant relationships with the Peaceful Acceptance subscale. Single individuals had significantly higher Peaceful Acceptance scores compared to married individuals (*p* = 0.045). Similarly, Peaceful Acceptance scores increased significantly with higher levels of education (*p* = 0.010), with the highest scores observed among participants with undergraduate and postgraduate degrees.

For the Struggle with Illness subscale, statistically significant differences were found in relation to chemotherapy status, recurrent/metastatic cancer, and living alone. Participants currently receiving chemotherapy had higher struggle scores than those who were not (*p* = 0.023). Likewise, participants with recurrent cancer or metastasis reported significantly higher struggle scores (*p* = 0.001). Additionally, those living alone also showed significantly higher levels of struggle (*p* = 0.030), suggesting that social isolation may contribute to a greater psychological burden during illness.

Although not statistically significant, a marginal difference was observed in the total PEACE score by age group (*p* = 0.054) and chemotherapy status (*p* = 0.059), indicating a potential trend that may reach significance with a larger sample size. In both cases, younger individuals and those currently receiving chemotherapy tended to report higher total PEACE scores.

Other demographic characteristics, such as gender (*p* = 0.118 for Peaceful Acceptance, *p* = 0.189 for Struggle with Illness, *p* = 0.702 for PEACE total score) and marital status or living status in relation to other subscales and total scores (*p* = 0.242 for Peaceful Acceptance, *p* = 0.882 for total PEACE score in living status), were not significantly associated with PEACE outcomes.

## Discussion

This study evaluates the validity and reliability of the Turkish version of the Peace, Equanimity, and Acceptance in the Cancer Experience (PEACE) scale, demonstrating that it provides a valid and reliable tool to measure acceptance and coping mechanisms for cancer patients in Turkey. The findings confirm that the PEACE scale is culturally adaptive, and its two-factor structure (Peaceful Acceptance and Coping with Illness) remains consistent across different cultures. This aligns with findings from previous international studies and supports the cross-cultural validity of the PEACE scale.

The structural validity of the Turkish PEACE scale was confirmed by exploratory factor analysis (EFA). The two-factor structure explained 53.16% of the total variance, indicating that the scale accurately measures acceptance and coping mechanisms culturally. When the original English version of the scale was developed by Mack et al. (2008), it was reported to have a two-factor structure, and Cronbach’s alpha coefficients ranged between 0.82 and 0.89. These findings show that the internal consistency of the scale is quite strong (Joa et al., 2020). In our study, Cronbach’s alpha values for the Turkish version were found to be in the range of 0.78–0.78 and were found to be compatible with the results of the original study (Mack et al., 2008). In the German version of the study, it was reported that the factor structure of the scale was confirmed, and very high statistical reliability, such as test-retest reliability (*r* = 0.920), was obtained. The test-retest correlation (*r* = 0.916) was similarly high in our Turkish study, indicating the consistency of the scale over time. The concurrent validity of the Turkish PEACE scale was supported by its significant negative correlation with the Cancer-Related Negative Social Expectations Scale (CRNSES), indicating that the distress and hopelessness of cancer patients decreased as their acceptance and coping skills improved. These findings are in line with the Polish study, which reported that PEACE scores were inversely related to distress and hopelessness (Czerw et al., 2017).

The validation of the PEACE scale in different cultural contexts allows for a broader understanding of how acceptance and struggle with illness manifest among cancer patients worldwide. The Turkish version of the PEACE scale demonstrated a two-factor structure, Peaceful Acceptance and Struggle With Illness, consistent with its original English version (Mack et al., 2008) and subsequent adaptations in other languages. However, cultural variations in illness perception and coping mechanisms have led to slight differences in psychometric properties across different linguistic adaptations. The German version of the PEACE scale (PEACE-G), validated by Sauer et al. (2024), confirmed a similar two-factor structure with high internal consistency and test-retest reliability (*r* = 0.920). The study found that German cancer patients with higher peaceful acceptance reported better health-related quality of life (HRQoL) and lower psychological distress, similar to findings from the Turkish study. However, the German version emphasized existential aspects of acceptance more strongly than the Turkish version, potentially due to differences in philosophical and spiritual perspectives on illness.

In contrast, the Japanese version of the PEACE scale highlighted cultural distinctions in acceptance and struggle with cancer. The Japanese study found that “Peace and Tranquility” were more strongly represented in their factor structure, reflecting the deep cultural influence of mindfulness and Buddhist principles on acceptance. While the Turkish version retained the two-factor structure, differences in factor loadings suggest that Turkish patients may interpret acceptance through a religious and familial support lens rather than an individual existential perspective (Okamura et al., 2022).

The Polish adaptation of the PEACE scale (Czerw et al., 2017) similarly confirmed its two-factor structure but identified a stronger negative correlation between the struggle with illness and psychological well-being. Polish cancer patients who struggled more with their diagnosis reported significantly higher levels of hopelessness and distress, which aligns with findings from the Turkish validation. However, the Polish study suggested that social support mechanisms play a lesser role in coping, whereas Turkish patients often rely heavily on religious and familial support systems (Karahan et al., 2018).

The relationship between patient characteristics and PEACE subscales provides further insight into how different sociodemographic factors influence acceptance and struggle with illness. Notably, married individuals exhibited lower acceptance scores compared to single individuals (*p* = 0.045), suggesting that family responsibilities may impact the ability to find peace with the disease. Higher education levels were associated with increased acceptance scores (*p* = 0.010), a finding that aligns with studies indicating that education enhances coping skills and health literacy (Cha et al. 2019). Patients undergoing chemotherapy and those facing recurrent or metastatic cancer often report heightened struggle scores, reflecting the profound psychological burden associated with disease progression. The study by Huang et al. provides evidence that patients in advanced stages of cancer, particularly those undergoing chemotherapy, experience significant psychological distress. This distress is further intensified in cases of recurrence or metastasis, with statistically significant struggle scores reported (*p* = 0.001) (Huang et al 2024). Patients undergoing chemotherapy and those facing recurrent or metastatic cancer often report heightened struggle scores, reflecting the profound psychological burden associated with disease progression. The study by Huang et al. demonstrates that patients in advanced stages of cancer, particularly those receiving chemotherapy, experience considerable psychological distress. This distress is significantly intensified in cases of recurrence or metastasis, as reflected in statistically significant struggle scores. (*p* = 0.001) (Huang et al 2024).

The current study revealed a moderate positive correlation between the PEACE and CRNSES total scores (*r* = 0.438, *p* < .001) regarding the perception of negative social expectations among Turkish cancer patients. This finding supports the concurrent validity of the Turkish version of the PEACE scale. At first glance, the positive direction of this correlation may appear counterintuitive. However, it can be interpreted in several culturally meaningful ways. In Turkish society, where strong family bonds and social conformity are emphasized, individuals with higher awareness and acceptance of their illness may become more attuned to social stigma or experience increased emotional sensitivity to societal reactions (Karahan et al., 2018). Alternatively, patients who feel socially isolated or misunderstood may turn inward and develop stronger spiritual or existential coping mechanisms, such as peaceful acceptance, to maintain emotional balance. Similar dynamics were noted in the Japanese adaptation of the PEACE scale, where cultural emphasis on social harmony and internal reflection influenced scale performance (Okamura et al., 2022). This suggests that inner peace and social context are not independent but interactively shape patients’ psychological adjustment to illness. This correlation highlights the importance of evaluating both individual and social dimensions of coping in culturally sensitive psycho-oncology research. Future studies may explore the mediating role of social support or stigma in the relationship between acceptance and psychological distress.

The PEACE scale’s validity and reliability in the Turkish context underscore its potential for clinical applications. Healthcare professionals can utilize this tool to assess patients’ acceptance levels and psychological distress, thereby guiding psychosocial interventions to enhance well-being and quality of life. Given the cultural significance of spirituality and social support in Turkey, future research could explore the role of these factors in influencing acceptance and coping strategies among cancer patients (Okamura et al., 2022).

In conclusion, this study provides strong evidence for the psychometric robustness of the Turkish version of the PEACE scale. The scale can serve as a valuable instrument for assessing acceptance and struggle in Turkish cancer patients, enabling tailored psychological and supportive care interventions. Future studies with larger and more diverse samples are recommended to validate and generalize these findings further. These cross-cultural comparisons indicate that, while the PEACE scale is a reliable and valid tool across different populations, cultural differences impact how cancer patients experience and express acceptance. The Turkish version, like its counterparts, confirms the crucial role of peaceful acceptance in reducing psychological distress. However, the importance of religious beliefs, family support, and social solidarity in Turkish culture appears to shape the interpretation of acceptance in ways that differ from those observed in Western and East Asian contexts.

### Clinical implications

The strong associations between peaceful acceptance and psychological variables such as distress, resilience, psychological flexibility, and mindfulness highlight the critical role of acceptance in the psychological well-being of cancer patients. Consistent with previous studies, higher levels of acceptance are linked to enhanced emotional stability and improved quality of life, while being associated with reduced anxiety, depression, and hopelessness (Czerw et al., 2017; Quinto et al., 2022). Given these findings, assessing acceptance levels in cancer patients is essential for developing effective psychosocial interventions. The PEACE scale serves as a valuable tool for healthcare professionals to evaluate patients’ acceptance levels and tailor interventions accordingly. Acceptance-based interventions, such as Acceptance and Commitment Therapy (ACT), offer promising approaches to enhancing psychological flexibility and promoting well-being. ACT encourages patients to embrace their illness experience without avoidance, thereby fostering a more adaptive coping mechanism. Studies have demonstrated that ACT contributes to improved quality of life and reduced psychological distress in individuals facing chronic illnesses, including cancer (Sauer et al., 2024). Additionally, mindfulness-based interventions, which inherently promote an accepting attitude toward all experiences, have been shown to have positive effects on psychological resilience and emotional adjustment in cancer patients (Pinquart et al., 2006).

### Implication for practices

The research also has important implications for clinical practice. The Turkish PEACE scale enables health professionals to more effectively assess the acceptance levels and coping mechanisms of cancer patients, facilitating the design of more targeted psychosocial interventions. The scale is a valuable tool for developing appropriate psychological and social support strategies to improve the quality of life of cancer patients. Future research should examine the sensitivity of the PEACE scale to changes in coping strategies and its long-term effects on the psychological well-being of cancer patients. Furthermore, a more detailed examination of cultural differences in the dimensions of the scale may help to develop culturally appropriate intervention strategies (Ahn et al., 2023; Karahan et al., 2018).

### Limitations

This study has several limitations that should be considered when interpreting the results. First, the sample size was relatively small (n = 90), which may limit the generalizability of the findings to the broader population of cancer patients in Turkey. Future studies with larger sample sizes are recommended to validate these findings further. Second, the study was conducted at a single oncology outpatient clinic in Kayseri City Training and Research Hospital, which may not represent the diverse experiences of cancer patients in different regions or healthcare settings in Turkey. A multi-center study would provide a more comprehensive understanding of the PEACE scale’s applicability across various contexts. Third, the cross-sectional design of the study limits the ability to draw conclusions regarding causal relationships between peaceful acceptance and struggle with illness. Longitudinal studies are needed to explore how these constructs evolve over time and how they impact patient outcomes.

## Conclusion

In conclusion, the PEACE scale, the Turkish version of which is valid and reliable, can serve as an effective measurement tool to assess the acceptance levels of cancer patients and their ability to live a life at peace with the disease.

## Authorship Statement

ÖÖ, MS, HÇ, GT, HZ, and ED conceptualized and designed the study and conducted the intervention. MS and ÖÖ analyzed the data. HZ wrote the first draft of the article. All authors revised the manuscript for important intellectual content and approved the final submitted version to be published.
